# Poly-D,L-Lactic Acid Stimulates Angiogenesis and Collagen Synthesis in Aged Animal Skin

**DOI:** 10.3390/ijms24097986

**Published:** 2023-04-28

**Authors:** Seyeon Oh, Suk Bae Seo, Gunpoong Kim, Sosorburam Batsukh, Kuk Hui Son, Kyunghee Byun

**Affiliations:** 1 Functional Cellular Networks Laboratory, Lee Gil Ya Cancer and Diabetes Institute, Gachon University, Incheon 21999, Republic of Korea; seyeon8965@gmail.com (S.O.); sosorburam72@gmail.com (S.B.); 2 SeoAh Song Dermatologic Clinic, Seoul 05557, Republic of Korea; kddm7@naver.com; 3 VAIM Co., Ltd., Okcheon 29055, Republic of Korea; gunpkim@vaim.co.kr; 4 Department of Anatomy & Cell Biology, College of Medicine, Gachon University, Incheon 21936, Republic of Korea; 5 Department of Thoracic and Cardiovascular Surgery, Gachon University Gil Medical Center, Gachon University, Incheon 21565, Republic of Korea; 6 Department of Health Sciences and Technology, Gachon Advanced Institute for Health & Sciences and Technology (GAIHST), Gachon University, Incheon 21999, Republic of Korea

**Keywords:** aged skin, heat shock protein 90, angiogenesis, collagen, skin rejuvenation, Poly-D,L-Lactic acid filler

## Abstract

Angiogenesis promotes rejuvenation in multiple organs, including the skin. Heat shock protein 90 (HSP90), hypoxia-inducible factor-1 alpha (HIF-1α), and vascular endothelial growth factor (VEGF) are proangiogenic factors that stimulate the activities of phosphoinositide 3-kinase (PI3K), protein kinase B (AKT), and extracellular signal-regulated kinase 1/2 (ERK1/2). Poly-D,L-lactic acid (PDLLA), polynucleotide (PN), and calcium hydroxyapatite (CaHA) are dermal fillers that stimulate the synthesis of dermal collagen. However, it is not yet known whether these compounds promote angiogenesis, which leads to skin rejuvenation. Here, we evaluated whether PDLLA, PN, and CaHA stimulate angiogenesis and skin rejuvenation using H_2_O_2_-treated senescent macrophages and endothelial cells as an in vitro model for skin aging, and we used young and aged C57BL/6 mice as an in vivo model. Angiogenesis was evaluated via endothelial cell migration length, proliferation, and tube formation after conditioned media (CM) from senescent macrophages was treated with PDLLA, PN, or CaHA. Western blot showed decreased expression levels of HSP90, HIF-1α, and VEGF in senescent macrophages, but higher expression levels of these factors were found after treatment with PDLLA, PN, or CaHA. In addition, after exposure to CM from senescent macrophages treated with PDLLA, PN, or CaHA, senescent endothelial cells expressed higher levels of VEGF receptor 2 (VEGFR2), PI3K, phosphorylated AKT (pAKT), and phosphorylated ERK1/2 (pERK1/2) and demonstrated greater capacities for cell migration, cell proliferation, and tube formation. Based on the levels of 4-hydroxy-2-nonenal, the oxidative stress level was lower in the skin of aged mice injected with PDLLA, PN, or CaHA, while the tumor growth factor (TGF)-β1, TGF-β2, and TGF-β3 expression levels; the density of collagen fibers; and the skin elasticity were higher in the skin of aged mice injected with PDLLA, PN, or CaHA. These effects were greater in PDLLA than in PN or CaHA. In conclusion, our results are consistent with the hypothesis that PDLLA stimulates angiogenesis, leading to the rejuvenation of aged skin. Our study is the first to show that PDLLA, PN, or CaHA can result in angiogenesis in the aged skin, possibly by increasing the levels of HSP90, HIF-1α, and VEGF and increasing collagen synthesis.

## 1. Introduction

As skin ages, dermal thinning and decreased skin elasticity lead to skin sagging, a loss of skin volume, and the formation of wrinkles [1,2]. In addition, alterations to the structure and density of collagen fibers are a major trigger for dermal thinning [3,4]. The collagen fiber structure of facial skin is disorganized by aging, and fragmented forms of collagen fibers are accumulated [4]. Moreover, the ability of fibroblasts to synthesize collagen decreases alongside chronological aging [3]. Matrix metalloproteinases (MMPs), which destroy collagen and elastin fibers, are increased during aging [3,4]. By both decreased collagen synthesis and the increased destruction of collagen, the density of collagen fibers decreases during aging [3,4].

Several compounds are commonly used as dermal fillers to promote skin rejuvenation, including hyaluronic acid (HA), polynucleotide (PN), calcium hydroxylapatite (CaHA), and poly (lactic acid) (PLA) [5,6,7]. The broad term “PLA” refers to stereoisomeric PLA variants, including poly (D-lactic acid) (PDLA), poly (L-lactic acid) (PLLA), poly-D,L-lactic acid (PDLLA), and racemic PLA [8]. These commonly used dermal fillers stimulate collagen synthesis in the skin and augment skin volume [5,6,7,9] by inducing a low-grade granulomatous inflammatory reaction [10,11], which is often mediated by M1 macrophage-secreted proinflammatory cytokines, such as interleukin (IL)-1β, IL-6, and tumor necrosis factor-α (TNF-α) [12]. This acute inflammatory response leads to tissue remodeling accompanied by increased polarization of M2 macrophages and increased IL-10 [13]. M2 macrophages stimulate the migration of fibroblasts to the surface of the filler and induce the secretion of transforming growth factor-β (TGF-β), which activates fibroblasts and triggers the synthesis of collagen [14,15].

Injectable PLLA dermal filler (Sculptra; Dermik Laboratories, Bridgewater, The Netherlands) was approved by the FDA for use in the treatment of HIV-associated facial lipoatrophy [16]. Its application was expanded to the cosmetic industry in 2009 [17]. Many animal and human studies have shown that PLLA filler increased collagen synthesis [18,19,20,21]. PDLLA filler is also biodegradable and biocompatible, which is similar to PLLA, and it increases collagen synthesis [6]. However, PDLLA is composed of spongiform microspheres and includes multiple micropores, while PLLA is composed of irregular solid microparticles [6,22]. A previous animal study showed that the abundance of type I collagen is higher between and inside PDLLA microspheres [6]. It has also been reported that PLLA induces subcutaneous nodules or papules, while these complications occur less often with PDLLA [23,24,25,26,27,28,29]. PN from salmon germ cells has been reported to stimulate fibroblasts, leading to increased collagen synthesis and greater skin elasticity [5]. However, PN fillers are less durable than PLA fillers [5]. CaHA is composed of calcium and phosphate and forms biodegradable, smooth-surfaced microspheres [30]. CaHA provides a substrate for fibroblast adhesion that stimulates collagen synthesis and deposition for at least 9 months [31,32].

Angiogenesis is also important for skin rejuvenation. The capacity for angiogenesis and the density of blood vessels in the skin decrease with aging [33,34,35]. Similarly, the abundance of growth factors that stimulate angiogenesis in the skin, including TGF-β, vascular endothelial growth factor (VEGF), platelet-derived growth factor (PDGF), and insulin-like growth factor-1 (IGF-1), declines with age [34,35,36,37,38,39]. Consistent with this, the intradermal injection of VEGF-loaded nanoparticles promotes skin rejuvenation [40]. Aging is also associated with increased oxidative stress resulting from the unbalanced formation and elimination of reactive oxidative species (ROSs) [41,42]. High levels of ROSs suppress levels of TGF-β or connective tissue growth factor (CTGF) and increase the abundance of MMPs, which leads to the decreased expression of type I collagen in the skin [43,44]. Loss of tissue perfusion is a primary factor leading to high levels of ROSs [45]. Some dermal fillers also induce the neovascularization of newly generated skin [46,47]. As newly generated vessels can increase blood supply and enhance the delivery of nutrients and oxygen to remodeled tissue, neovascularization promotes wound repair and tissue regeneration [46,47]. CaHA also stimulates the expression of CD34, potentially leading to increased angiogenesis [47]. While CaHA showed increased angiogenesis in a skin biopsy, the mechanism of how CaHA increases angiogenesis has not yet been revealed. Moreover, PDLLA and PN are known to stimulate the synthesis of dermal collagen; however, their impact on skin rejuvenation via angiogenesis has not been thoroughly studied. To address this knowledge gap, the present study investigated the effects of PDLLA on angiogenesis, oxidative stress, and collagen synthesis.

The primary inducer of VEGF expression is hypoxia-inducible factor-1 α (HIF-1α), which is primarily expressed in macrophages, fibroblasts, endothelial cells, and keratinocytes [48,49,50,51,52,53,54]. The expression of heat shock proteins (HSPs), which are essential for cellular homeostasis, increases in response to various external stimuli and intracellular stress [55,56]. HSPs also increase during acute and chronic inflammation and in response to circulating cytokines [54,57]. The binding of HSP90 to HIF-1α increases its activity by inhibiting the von Hippel–Lindau protein (VHL)-independent proteasomal degradation of HIF-1α and increases HIF-1α-mediated gene transcription [58]. VEGF initiates angiogenesis primarily by binding to and activating VEGF receptor 2 (VEGFR2) [59,60,61]. Activated VEGFR2 upregulates downstream signaling pathways, including those regulated by phosphoinositide 3-kinase (PI3K), protein kinase B (AKT), and extracellular signal-regulated kinase 1/2 (ERK1/2) [62,63,64,65]. These pathways stimulate the proliferation, migration, and survival of endothelial cells [62,63,64,65], and ERK1/2 signaling promotes the formation of vessel-like endothelial cell tubes during angiogenesis [66].

We hypothesized that dermal fillers, including PDLLA, PN, and CaHA, induce a mild inflammatory response, leading to increased levels of HSP90, HIF-1α, and VEGF in the skin. Furthermore, we hypothesized that VEGF then upregulates the PI3K, AKT, and ERK1/2 signaling pathways, leading to enhanced neovascularization, decreased ROS levels, increased TGF-β levels, and the increased synthesis of collagen. The first purpose of this study was to evaluate whether PDLLA, PN, and CaHA increased angiogenesis, which contributed to increasing collagen density in aged skin. The second purpose was to study which signal pathway involved in angiogenesis was activated by PDLLA, PN, and CaHA. To evaluate these, we used young and aged male C57BL/6 mice as an in vivo model for animal skin aging. Moreover, we made relevant in vitro aging models with H_2_O_2_-treated senescent macrophages. Our finding showed that PDLLA increased angiogenesis by increasing HSP90, HIF-1α, and VEGF, which was accompanied with increased collagen fiber accumulation in aged skin.

## 2. Results

### 2.1. PDLLA Increases Expression of HSP90, HIF-1α, and VEGF in Senescent Macrophages and the Skin of Aged Mice

**Figure 1:**
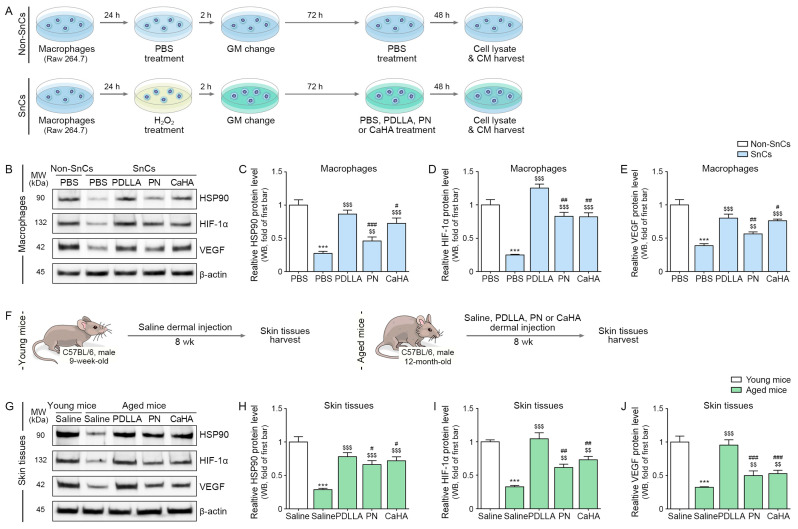
Upregulation of HSP90, HIF-1α, and VEGF via PDLLA in senescent macrophages and aged skin. (**A**) Schematic diagram of experimental design including induction of senescence with H_2_O_2_ and treatment of senescent macrophages with PDLLA, PN, or CaHA. Before harvesting cells, supernatants (CM) of macrophages were collected. Proteins were isolated from cell lysates; (**B**) expression of HSP90, HIF-1α, and VEGF in macrophages was confirmed via Western blot; (**C**–**E**) the graphs show quantification of the Western blot data in (**B**). Expression levels of HSP90, HIF-1α, and VEGF decreased after treatment with senescent cells and increased after treatment with PDLLA, PN, or CaHA in senescent cells; (**F**) 9-week-old male C57BL/6 mice were injected with saline (100 μL total over 5 injection sites) into the dermal layer of the back. Male 12-month-old mice were injected with saline, PDLLA, PN, or CaHA (100 μL total over 5 injection sites) into the dermal layer of the back. Skin samples were collected 8 weeks after injection; (**G**) expression of HSP90, HIF-1α, and VEGF in young or aged skin was confirmed via Western blot; (**H**–**J**) the graphs show quantification of the Western blot data shown in (**G**). HSP90, HIF-1α, and VEGF levels were lower in aged mice injected with saline and higher in aged mice injected with PDLLA, PN, or CaHA. To correct for differences in protein loading, the quantification of the Western blot was normalized using β-actin as a loading control protein. For each blot, the values were expressed relative to the mean of the first group. Data are presented as mean ± SD (*n* = 3/group). ***, *p* < 0.001, first bar vs. second bar; $$ and $$$, *p* < 0.01 and *p* < 0.001, second bar vs. third, fourth, or fifth bar; #, ##, and ###, *p* < 0.05, *p* < 0.01, and *p* < 0.001, third bar vs. fourth or fifth bar. β-actin, beta-actin; CaHA, calcium hydroxyapatite; CM, conditioned medium; GM, growth medium; HIF-1α, hypoxia-inducible factor-1 alpha; h, hours; HSP90, heat shock protein 90; kDa, kilodalton; MW, molecular weight; PBS, phosphate-buffered saline; PDLLA, poly-D,L-lactic acid; PN, polynucleotide; SD, standard deviation; SnCs, senescent cells; VEGF, vascular endothelial growth factor; WB, Western blot; wk, weeks.

First, we evaluated whether PDLLA increased the expression levels of HSP90 in H_2_O_2_-treated senescent macrophages. This experiment was performed with H_2_O_2_-treated senescent macrophages because this is the most widely used in vitro model for skin aging [67]. Briefly, murine monocyte/macrophage Raw 264.7 cells were exposed to phosphate-buffered saline (PBS) or H_2_O_2_ for 2 h and then treated with PBS, PDLLA, PN, or CaHA (Figure 1A). In comparison to non-senescent macrophages, HSP90, HIF-1α, and VEGF were expressed at lower levels (0.27-, 0.26-, or 0.39-fold, respectively) in senescent cells. However, treatment with PDLLA, PN, or CaHA (HSP90: 3.31-, 1.74-, or 2.78-fold, respectively; HIF-1α: 4.66-, 3.17-, or 3.05-fold, respectively; VEGF: 2.07-, 1.45-, or 1.97-fold, respectively) increased the abundance of these factors, with PDLLA having the strongest effect (Figure 1B–E). HSP90, HIF-1α, and VEGF were expressed at lower levels (0.28-, 0.33-, or 0.34-fold, respectively) in the skin of aged mice than in the skin of young mice. The levels of these proteins were higher in the skin of aged mice injected with PDLLA, PN, or CaHA (HSP90: 2.75-, 2.33-, or 2.71-fold, respectively; HIF-1α: 3.19-, 1.88-, or 2.54-fold, respectively; VEGF: 2.8-, 1.48-, or 1.57-fold, respectively) than in the skin of aged mice (Figure 1F–J). The most prominent effect was in mice injected with PDLLA.

### 2.2. PDLLA Upregulated VEGFR2, PI3K, pAKT/AKT, and pERK1/2/ERK1/2 in the Senescent Endothelial Cells and Aged Skin

**Figure 2:**
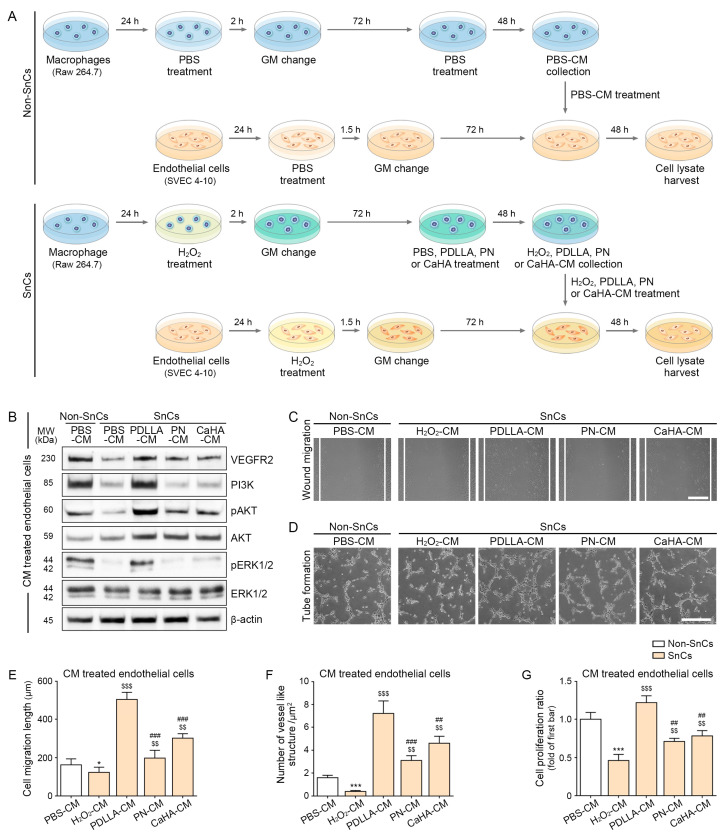
Upregulation of angiogenesis pathways in senescent endothelial cells exposed to CM from PDLLA-treated senescent macrophages. (**A**) Schematic diagram showing treatment of H_2_O_2_-treated senescent endothelial cells with CM. Proteins were isolated from cell lysates; (**B**) expression of VEGFR2, PI3K, pAKT, AKT, pERK1/2, and ERK1/2 in CM-treated endothelial cells was confirmed via Western blot. Quantitation of the Western blot in (**B**) is presented in Figure S1. Expression levels of VEGFR2, PI3K, pAKT/AKT, and pERK1/2/ERK1/2 decreased in cells exposed to H_2_O_2_/PBS-CM and increased in cells exposed to H_2_O_2_/PDLLA-CM, PN-CM, or CaHA-CM. To correct for differences in protein loading, the quantification of the Western blot was normalized using β-actin as a loading control protein. For each blot, the values were expressed relative to the mean of the first group; (**C**) representative images show the results of cell migration assay using endothelial cells exposed to CM (scale bar = 500 µm); (**D**) representative images show the results of the tube formation assay using endothelial cells exposed to CM (scale bar = 500 µm); (**E**) quantitation of migration assay results shown in (**C**). Migrating distances were shorter in cells exposed to H_2_O_2_/PBS-CM and longer in cells exposed to H_2_O_2_/PDLLA-CM, PN-CM_,_ or CaHA-CM; (**F**) quantitation of tube formation assay results shown in (**D**). The number of vessel-like tube structures was decreased in cells exposed to H_2_O_2_/PBS-CM and increased in cells exposed to H_2_O_2_/PDLLA-CM, PN-CM_,_ or CaHA-CM; (**G**) cell proliferation decreased in senescent endothelial cells exposed to H_2_O_2_/PBS-CM and increased in senescent endothelial cells exposed to H_2_O_2_/PDLLA-CM, PN-CM, or CaHA-CM. Data are presented as mean ± SD (*n* = 3/group). * and ***, *p* < 0.05 and *p* < 0.001, first bar vs. second bar; $$ and $$$, *p* < 0.01 and *p* < 0.001, second bar vs. third, fourth, or fifth bar; ## and ###, *p* < 0.01 and *p* < 0.001, third bar vs. fourth or fifth bar. AKT, protein kinase B; β-actin, beta-actin; CaHA, calcium hydroxyapatite; CM, conditioned medium; ERK1/2, extracellular signal-regulated kinase 1/2; GM, growth medium; h, hours; kDa, kilodalton; MW, molecular weight; pAKT, phosphorylated AKT; PBS, phosphate-buffered saline; PDLLA, poly-D,L-lactic acid; pERK1/2, phosphorylated ERK1/2; PI3K, phosphoinositide 3-kinase; PN, polynucleotide; SD, standard deviation; SnCs, senescent cells; VEGFR2, vascular endothelial growth factor receptor 2.

We hypothesized that PDLLA induces the expression of HSP90, HIF-1α, and VEGF in macrophages and that VEGF secreted by macrophages subsequently stimulates angiogenesis in endothelial cells. To test this, conditioned media (CM) from senescent macrophages treated with PBS, PDLLA, PN, or CaHA were collected and administered to PBS- or H_2_O_2_-treated SEVC4-10 endothelial cells (Figure 2A). In the treatment with CM from H_2_O_2_/PBS-treated macrophages, the VEGFR2 expression level was lower in senescent endothelial cells (0.35-fold). In contrast, the expression level of VEGFR2 increased in senescent cells treated with CM from H_2_O_2_/PDLLA-, H_2_O_2_/PN-, or H_2_O_2_/CaHA-treated macrophages (2.36-, 1.64-, or 1.6-fold, respectively), with the most prominent effect being observed with CM from H_2_O_2_/PDLLA-treated macrophages (Figure 2B and Figure S1A). In the treatment with CM from H_2_O_2_/PBS-treated macrophages, the level of PI3K expression was lower in senescent endothelial cells (0.27-fold). However, the expression levels of PI3K increased in senescent endothelial cells exposed to CM from macrophages treated with PDLLA (3.98-fold; Figure 2B and Figure S1B).

The ratios of phosphorylated AKT to total AKT (pAKT/AKT) and of phosphorylated ERK1/2 to total ERK1/2 (pERK1/2/ERK1/2) were lower in endothelial cells exposed to CM from H_2_O_2_/PBS-treated macrophages (0.24- and 0.11-fold, respectively). However, the ratio of pAKT/AKT increased after exposure to CM from H_2_O_2_/PDLLA-, H_2_O_2_/PN-, or H_2_O_2_/CaHA-treated macrophages (4.41-, 2.03-, or 2.08-fold, respectively). Additionally, the ratio of pERK1/2/ERK1/2 increased after exposure to CM from H_2_O_2_/PDLLA- and H_2_O_2_/CaHA-treated macrophages (10.14- and 2.08-fold, respectively). These ratios represent the most prominent effects of the treatment with CM from H_2_O_2_/PDLLA-treated macrophages (Figure 2B and Figure S1C,D).

A scratch wound assay was used to evaluate the migratory capacity of endothelial cells exposed to CM. The results show that shorter migration distances were caused by senescent endothelial cells exposed to CM from H_2_O_2_/PBS relative to non-senescent cells exposed to CM from PBS-treated macrophages (0.76-fold), but longer migration distances were observed after exposure to CM from H_2_O_2_/PDLLA-, H_2_O_2_/PN-, or H_2_O_2_/CaHA-treated macrophages (4.5-, 1.94-, or 2.88-fold, respectively; Figure 2C,E). The most prominent effect was observed in cells exposed to CM from H_2_O_2_/PDLLA-treated macrophages.

The tube formation capacity was also examined using endothelial cells cultured on Matrigel. The number of vessel-like structures was lower in the treatment with senescent endothelial cells exposed to CM from H_2_O_2_/PBS-treated macrophages than that with non-senescent cells exposed to CM from PBS-treated macrophages (0.25-fold), but the number was higher after exposure to CM from H_2_O_2_/PDLLA-, H_2_O_2_/PN-, or H_2_O_2_/CaHA-treated macrophages (18-, 7.75-, or 11.5-fold, respectively; Figure 2D,F). The most prominent effect was observed with cells treated with CM from H_2_O_2_/PDLLA-treated macrophages.

The rate of endothelial cell proliferation was lower after exposure to CM from H_2_O_2_/PBS-treated macrophages than with non-senescent cells exposed to CM from PBS-treated macrophages (0.46-fold), but the cell proliferation rate increased after exposure to CM from H_2_O_2_/PDLLA-, H_2_O_2_/PN-, or H_2_O_2_/CaHA-treated macrophages (2.65-, 1.54-, or 1.7-fold; Figure 2G). The most prominent effect was observed with CM from H_2_O_2_/PDLLA-treated macrophages.

The quantitative Western blot analysis of VEGFR2, PI3K, pAKT/AKT, and pERK1/2/ERK1/2 indicated lower expression levels in the skin of aged mice than in young mice (0.07-, 0.36-, 0.24-, and 0.07-fold), but the VEGFR2, PI3K, and pAKT/AKT ratios were expressed at higher levels in the skin of aged mice injected with PDLLA, PN, or CaHA (VEGFR2: 14.44-, 3.63-, or 7.0-fold, respectively; PI3K: 2.78-, 2.20-, or 1.14-fold, respectively; pAKT/AKT: 4.6-, 2.17-, or 2.29-fold, respectively). The ratio of pERK1/2/ERK1/2 was increased in the skin of aged mice injected with PDLLA (8.42-fold; Figure S2). The most prominent increases were observed in aged mice injected with PDLLA.

### 2.3. Injection of Aged Mice with PDLLA Decreases Oxidative Stress and Increases TGF-β

**Figure 3:**
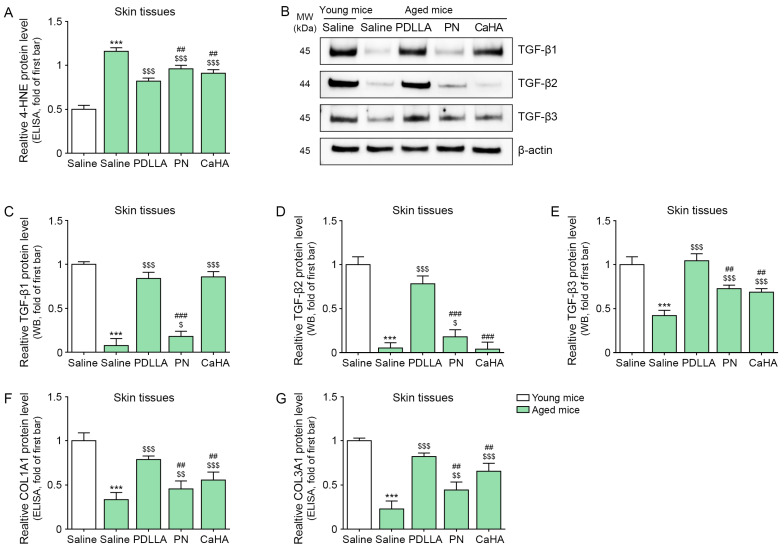
Upregulation of TGF-β and collagen and reduced oxidative stress in fibroblasts of aged mice injected with PDLLA. (**A**) Expression level of 4-HNE (oxidative stress marker) in the skin of aged mice was measured via ELISA. Expression level of 4-HNE was higher in aged mice injected with saline and lower in aged mice injected with PDLLA, PN, or CaHA; (**B**) expression of TGF-β1, -β2, and -β3 in aged skin was confirmed via Western blot; (**C**–**E**) quantitation of Western blot data shown in (**B**). TGF-β1, -β2, and -β3 levels were lower in aged mice injected with saline and higher in aged mice injected with PDLLA, PN, or CaHA. To correct for differences in protein loading, the quantification of the Western blot was normalized using β-actin as a loading control protein. For each blot, the values were expressed relative to the mean of the first group; (**F**,**G**) expression level of COL1A1 and COL3A1 in aged skin was confirmed via ELISA. COL1A1 and COL3A1 were expressed at lower levels in aged mice injected with saline but were expressed at higher levels in aged mice injected with PDLLA, PN, or CaHA. Data are presented as mean ± SD (*n* = 3/group). ***, *p* < 0.001, first bar vs. second bar; $, $$, and $$$, *p* < 0.05, *p* < 0.01, and *p* < 0.001, second bar vs. third, fourth, or fifth bar; ## and ###, *p* < 0.01 and *p* < 0.001, third bar vs. fourth or fifth bar. 4-HNE, 4-hydroxy-2-nonenal; β-actin, beta-actin; CaHA, calcium hydroxyapatite; COL1A1, collagen type 1a1; COL3A1, collagen type 3a1; kDa, kilodalton; MW, molecular weight; PDLLA, poly-D,L-lactic acid; PN, polynucleotide; SD, standard deviation; TGF-β, tumor growth factor-beta; WB, Western blot.

4-hydroxy-2-nonenal (4-HNE) was measured as a marker of oxidative stress [68] in the skin of young and aged mice. Our results show that there were higher levels of 4-HNE in the skin of aged mice than in young mice (2.32-fold). However, the level of 4-HNE decreased when aged mice were injected with PDLLA, PN, or CaHA (0.71-, 0.83-, or 0.79-fold, respectively; Figure 3A). The largest decrease was observed after the mice were injected with PDLLA.

TGF-β exists in three isoforms, TGF-β1, -β2, and -β3, all of which play roles in cell differentiation, cell migration, and transcription [69]. The expression level of TGF-β1 was lower in the skin of aged mice than in young mice (0.1-fold) but it increased after aged mice were injected with PDLLA, PN, or CaHA (8.84-, 2.08-, or 9.06-fold, respectively). The size of the increase was similar after the mice were injected with PDLLA or CaHA (Figure 3B,C). The expression level of TGF-β2 was lower in the skin of aged mice than in young mice (0.05-fold) but it increased after aged mice were injected with PDLLA or PN (16.12- or 3.65-fold; Figure 3B,D). Additionally, the expression level of TGF-β3 was increased after aged mice were injected with PDLLA, PN, or CaHA (2.34-, 1.67-, or 1.59-fold, respectively; Figure 3B,E). A similar expression pattern and response to PDLLA was observed for collagen (COL) 1A1 and COL3A1 (Figure 3F,G).

### 2.4. Injection of Aged Mice with PDLLA Increases Dermal Collagen, Dermal Thickness, and Skin Elasticity

**Figure 4:**
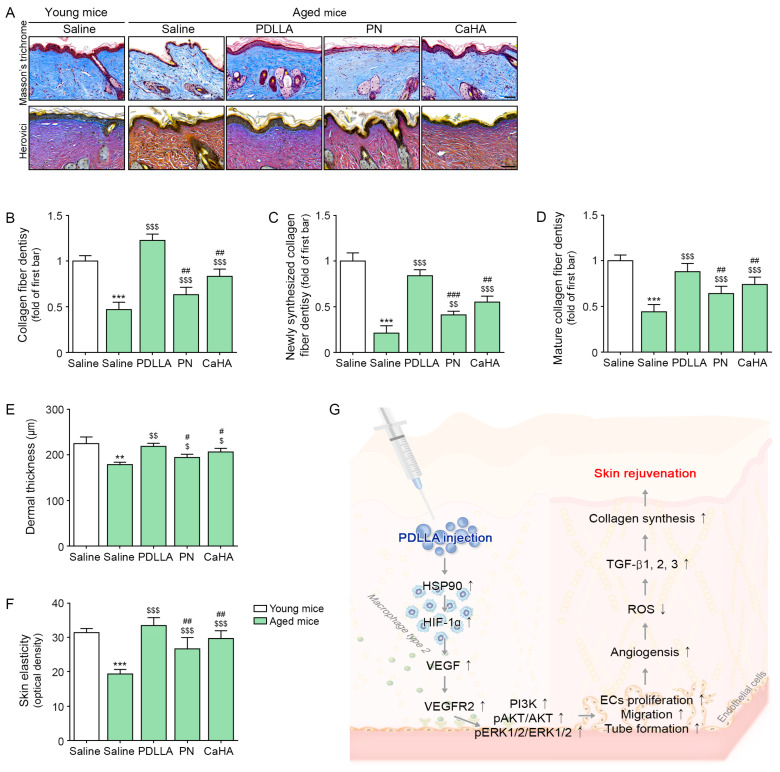
Upregulated effect of PDLLA on collagen fibers in aged skin. (**A**) Masson’s trichrome and Herovici’s staining in aged skin (scale bar = 100 µm); (**B**–**D**) quantification of the data in (**A**). The densities of collagen fibers (**B**), newly synthesized collagen fibers (**C**), and mature collagen fibers (**D**) were lower in aged than in young skin but increased in aged mice injected with PDLLA, PN, or CaHA; (**E**) the dermal thickness was lower in aged mice than in young mice but it increased in aged mice injected with PDLLA, PN, or CaHA. (**F**) Skin elasticity was lower in aged skin than in young skin but increased in aged mice injected with PDLLA, PN, or CaHA. Data are presented as mean ± SD (*n* = 3/group); (**G**) summary of the study. ** *p* < 0.01, ***, *p* < 0.001, first bar vs. second bar; $, $$, and $$$, *p* < 0.05, *p* < 0.01 and *p* < 0.001, second bar vs. third, fourth, or fifth bar; #, ##, and ###, *p* < 0.05, *p* < 0.01, and *p* < 0.001, third bar vs. fourth or fifth bar. AKT, protein kinase B; CaHA, calcium hydroxyapatite; ECs, endothelial cells; ERK1/2, extracellular signal-regulated kinase 1/2; HIF-1α, hypoxia-inducible factor-1 alpha; HSP90, heat shock protein 90; pAKT, phosphorylated AKT; PDLLA, poly-D,L-lactic acid; pERK1/2, phosphorylated ERK1/2; PI3K, phosphoinositide 3-kinase; PN, polynucleotide; ROS, reactive oxidative species; SD, standard deviation; TGF-β, tumor growth factor-beta; VEGF, vascular endothelial growth factor; VEGFR2, VEGF receptor 2.

The density of collagen fibers was evaluated using Masson’s trichrome staining method. The results showed that the collagen density was lower in the skin of aged mice than in young mice (0.47-fold). However, the collagen density increased when aged mice were injected with PDLLA, PN, or CaHA (2.62, 1.35, or 1.78, respectively), most prominently after injection with PDLLA (Figure 4A,B). Herovici’s staining was also used to differentiate newly formed collagen (blue) from mature collagen (red) [70,71]. This analysis showed that the densities of both newly synthesized and mature collagen were lower in the skin of aged than in young mice (0.21- and 0.44-fold) but the injection of PDLLA, PN, or CaHA increased the densities of both newly synthesized (4.0-, 1.95-, or 2.62-fold, respectively) and mature collagen (2.0-, 1.45-, or 1.68-fold, respectively) in aged mice, with the greatest effect being observed after injection with PDLLA (Figure 4A,C,D). The dermal thickness was lower in aged mice than in young mice (0.8-fold) but it increased in aged mice injected with PDLLA, PN, or CaHA (1.22-, 1.09-, or 1.15-fold, respectively), with the largest effect being observed after mice were injected with PDLLA (Figure 4E). Similar results were observed when skin elasticity was measured in young and aged mice using the API-100 Skin Analysis Machine (Aram Huvis, Republic of Korea). The skin elasticity was lower in aged mice than in young mice (0.62-fold) but it increased in aged mice injected with PDLLA, PN, or CaHA, with the largest effect being observed after injection with PDLLA (1.73-, 1.38-, or 1.53-fold, respectively; Figure 4F).

## 3. Discussion

Based on the “angiogenesis hypothesis of aging”, proangiogenic therapies could attenuate or slow the aging process because the expression levels of angiogenic factors and capillary density decline with age [72,73]. Consistent with this idea, a recent study has shown that VEGF-A promotes organ rejuvenation [40] and it was found that dermal thickness and collagen fiber thickness in old human skin increased after transplantation to a young mouse but did not increase after transplantation to an old mouse [40]. Furthermore, the abundance of CD31-positive endothelial cells and VEGF-A in old human skin increased after transplantation to a young mouse [40]. While angiogenesis is thought to be essential for skin rejuvenation, little is known regarding the effect of dermal fillers on angiogenesis. A previous study, in which CaHA was injected in the neck area of 21 women, showed that CD34 expression in the skin was increased both 4 months and 7 months after the injection [47]. Immunohistochemical staining results showed increased type I collagen intensity 4 months after the injection, which was accompanied by increased dermal thickness [47]. The authors suggested that increased angiogenesis markers could improve blow flow and increase nutrition supply in the filler injection site [47]. However, this study did not evaluate what the mechanism of CaHA-induced angiogenesis was.

PDLLA is also a bio-stimulating dermal filler. When PDLLA was injected into 2-week-old Sprague Dawley rats, there were no abnormalities in the injection sites until the 20th week [6]. Myofibroblasts appeared from the 2nd week and their number increased until the 20th week [6]. Type I collagen fiber around microspheres was observed from 4 weeks after the injection [6]. The authors also observed vessel-like structures in the PDLLA injection site from 2 weeks after the injection [6]; however, they did not pay much attention to or explain the meaning of angiogenesis.

PN fillers were also shown to increase the migration and type I collagen synthesis of human dermal fibroblasts [74]. The injection of PN filler into 6-week-old mice resulted in increased collagen fiber abundance 12 and 24 weeks after the injection [74]. Polydeoxyribonucleotide is known to increase cell activity, provide anti-inflammation, and cause collagen synthesis [75]. Thus, it has been used as an antiaging agent or in skin-regeneration materials [75]. While polydeoxyribonucleotide has been reported to increase angiogenesis, it has not been evaluated whether PN filler increases angiogenesis.

Previous studies have indicated that the HIF-1α-dependent expression of VEGF occurs primarily under hypoxic conditions [48,49,50,51,52]. It was shown that polydeoxyribonucleotide promotes wound healing by increasing the levels of HSP70, HSP90, and TGF-β [76]. Moreover, polydeoxyribonucleotide was shown to increase HIF-1α levels and decrease the prevalence of ischemic-reperfusion injury [77]. However, it has not been fully reported whether PDLLA or CaHA increases the levels of HSPs or HIF-1α. Since various stress conditions, such as heat, oxidative stress, acute or chronic inflammatory diseases, viral or bacterial infections, hypoxic conditions, heavy metals, and exercise, are known to increase HSPs [78,79,80,81], it is possible that PDLLA or CaHA lead to increased levels of HSPs via the promotion of mild inflammation. As HSP90 stimulates HIF-1α activity, we investigated whether PDLLA, PN, or CaHA induces HIF-1α via HSP90. Moreover, reactions to foreign bodies are typically mediated by macrophages; in these experiments, we used H_2_O_2_-treated senescent macrophages as an in vitro model for skin aging. HSP90, HIF-1α, and VEGF were expressed at a lower level in senescent macrophages than in non-senescent macrophages, but their expression levels increased when senescent cells were treated with PDLLA, PN, or CaHA. Furthermore, exposure to CM from H_2_O_2_/PDLLA-, H_2_O_2_/PN-, or H_2_O_2_/CaHA-treated macrophages increased the expression levels of VEGFR2, PI3K, phosphorylated AKT, and phosphorylated ERK1/2 in senescent endothelial cells but treatment with CM from H_2_O_2_/PBS-treated macrophages did not. As expected, after exposure to CM from H_2_O_2_/PDLLA-, H_2_O_2_/PN-, or H_2_O_2_/CaHA-treated macrophages, senescent endothelial cells showed greater capacities for the migration, proliferation, and formation of vessel-like tube structures. These results are consistent with the idea that treatment with PDLLA, PN, or CaHA causes the levels of HSP90, HIF-1α, and VEGF to increase in senescent macrophages and, in turn, secreted VEGF upregulates angiogenesis signaling pathways in endothelial cells. The angiogenesis effect of dermal fillers has not been evaluated with an in vitro model. Our study firstly showed that dermal fillers could induce angiogenesis, possibly via increasing HSP90, HIF-1α, and VEGF with in vitro models of senescent macrophage and endothelial cells. Even though macrophages’ involvement in stimulating fibroblasts to promote collagen synthesis has been considered one of the main mechanisms of dermal filler, a significant number of previous studies evaluated collagen synthesis with fibroblasts [6,47,74]. In contrast to previous studies, we hypothesized that we would observe increased angiogenesis mainly via macrophages’ modulation of dermal fillers. Thus, we examined endothelial cells exposed to conditioned media treated with macrophage cultures to evaluate their angiogenesis ability.

Similar results were obtained in our in vivo model for skin aging using young and aged male C57BL/6 mice. HSP90, HIF-1α, and VEGF were expressed at lower levels in the skin of aged mice than in the skin of young mice, but these levels increased after aged mice were injected with PDLLA, PN, or CaHA. Consistent with this, angiogenesis-related signaling mediated by VEGFR2, PI3K, AKT, and ERK1/2 increased in the skin of aged mice injected with PDLLA, PN, or CaHA. As decreased tissue perfusion leads to increased oxidative stress [45], we postulated that increased angiogenesis could stimulate tissue perfusion and attenuate oxidative stress in the injected mice. Consistent with this, the level of 4-HNE was lower in the skin of aged mice injected with PDLLA, PN, or CaHA.

Tissue destruction by ROSs is the main pathophysiology of aging [82]. Increased ROS levels lead to decreases in fibroblast size and the TGF-β/Smad pathway, which eventually cause decreased levels of extracellular matrix protein synthesis [82,83,84]. In the present study, the levels of TGF-β1, -β2, and -β3 were higher in old mice injected with PDLLA, PN, or CaHA than in the control mice. TGF-β isoforms are essential factors for fibrosis; thus, the overexpression of TGF-β can lead to pathological fibrosis [85,86,87]. The overexpression of TGF-β1 is reported to induce hypertrophic scarring [88]. However, TGF-β3 can promote wound healing without hypertrophic scarring [89]. In the present study, the expression levels of all three isoforms of TGF-β increased, suggesting that PDLLA, PN, and CaHA might be able to stimulate collagen synthesis without the potential complication of pathological fibrosis. Lastly, the injection of older mice with PDLLA and, to a lesser extent, PN or CaHA increased collagen fiber density, skin elasticity, and dermal thickness. These results suggest that PDLLA could be the preferred dermal filling agent for the promotion of angiogenesis, leading to the rejuvenation of aged skin. Previous studies which showed dermal filler’s effect on increasing collagen synthesis have been generally performed with young animal models [6,74]. As the ability of fibroblasts to synthesize collagen fibers is decreased and oxidative stress is increased in aged tissue [82], the collagen synthesis caused by dermal filler could be different in young and aged skin. Our study showed that PDLLA, PN, and CaHA increased collagen accumulation, even in aged skin.

## 4. Materials and Methods

The experimental process is shown in Figures S3 and S4.

### 4.1. PDLLA Preparation

Approximately 60 g of PDLLA was dissolved in 1 L of ethylene carbonate (Sigma-Aldrich, St. Louis, MO, USA): dimethyl sulfoxide (Sigma-Aldrich, St. Louis, MO, USA) (1:9) and then sprayed into cold n-hexane (<−10 °C; Sigma-Aldrich, St. Louis, MO, USA). The resulting solvent/polymer mixture was added to distilled water (DW) and passed through a filter to separate the solvent from 10 to 30 µm of solid PDLLA. Solid PDLLA was dried and mixed with 0.6% HA solution at a ratio of 17:3. Then, aliquots of the mixture were lyophilized in 10 mL vials and sterilized with gaseous ethylene oxide before use.

### 4.2. In Vitro Experiments

#### 4.2.1. Cell Culture

Murine macrophages (RAW 264.7) were obtained from the Korea Cell Line Bank (Seoul, Republic of Korea), and murine endothelial cells (ECs; SVEC4-10) were obtained from the American Type Culture Collection (ATCC, Manassas, VA, USA). Macrophages and ECs were routinely cultured in Dulbecco’s modified Eagle medium (HyClone-Cytiva^TM^, Marlborough, MA, USA) supplemented with 10% fetal bovine serum (Gibco^TM^-Thermo Fisher Scientific, Rockford, IL, USA) and 1% penicillin/streptomycin (Welgene, Gyeongsan, Republic of Korea) at 37 °C in a humidified atmosphere with 5% CO_2_.

#### 4.2.2. PDLLA, PN, and CaHA Treatment of H_2_O_2_-Treated Senescent Macrophages

To induce the senescence of macrophages, the method of senescence induction was slightly modified as follows [67,90]. The growth medium (GM) treatment time after H_2_O_2_ treatment was tested using senescence markers (p21 and p16) to establish the optimal time needed to induce senescence (Figure S5). Accordingly, the macrophages were treated with 100 μM of H_2_O_2_ (Sigma-Aldrich, St. Louis, MO, USA) for 2 h, washed with Dulbecco’s phosphate-buffered saline (DPBS, Gibco^TM^-Thermo Fisher Scientific, Rockford, IL, USA), returned to fresh GM, and incubated for 72 h. Next, 400 µL of PDLLA, PN, or CaHA (VAIM Co. LTD, Okcheon, Republic of Korea) was added into the cells and incubated for 48 h. Then, the supernatants (conditioned medium; CM) were collected for the treatment of ECs and cell lysates were collected for protein or RNA extraction (Figure 1A).

#### 4.2.3. Effect of CM on Angiogenesis Pathways in H_2_O_2_-Treated Senescent Endothelial Cells

ECs were treated with 50 μM of H_2_O_2_ for 2 h, transferred to fresh GM, and incubated for 72 h [91]. Then, CM was diluted in an equal volume of GM, added to the cells, and incubated for 48 h. The cells were used for angiogenesis assays or harvested for protein expression analysis (Figure 2A).

### 4.3. In Vivo Model

#### 4.3.1. Animal Model and Maintenance

Male C57BL/6 mice (8-week-old and 11-month-old) were obtained from Orient Bio (Seongnam, Republic of Korea). The mice were housed in standard cages with free access to food and water and were maintained at controlled temperature (22 ± 5 °C), relative humidity (50 ± 10%), and a 12 h light/dark cycle. The experimental protocols were approved by the ethical board of the Center of Animal Care and Use and were conducted in accordance with the Institutional Animal Care and Use Committee of Gachon University (Approval Number: LCDI-2021-0156).

#### 4.3.2. Experimental Design

After acclimatization for one week, 9-week-old mice were assigned to the young group (*n* = 3) and 12-month-old mice were randomly assigned to the aged group (*n* = 3 per group).

Next, 100 µL of PDLLA, PN, or CaHA was injected into the dermal layer on the back of each mouse using a syringe (KOREAVACCINE, Seoul, Republic of Korea). The total volume was distributed over five injection points. The control mice were injected with saline. After 8 weeks of injection, the mice were euthanized using respiratory anesthesia (isoflurane; HANA Pharm Co., Ltd., Seoul, Republic of Korea) and the injection sites were shaved. Then, skin elasticity was measured using a skin analyzer (AramHubis, Seoul, Republic of Korea) and skin samples were collected (Figure 1F).

### 4.4. Sample Preparation

#### 4.4.1. RNA Extraction and cDNA Synthesis

The RNA of the cell lysates (1 × 10^6^ cells per mL) was lysed in an RNAiso reagent (TAKARA, Tokyo, Japan) according to the manufacturer’s instructions. Briefly, lysed samples were mixed with chloroform (Samchun, Seoul, Republic of Korea) and centrifuged at 12,000× *g* for 15 min at 4 °C to separate the aqueous phase containing the RNA. The aqueous phase was transferred to a fresh tube, and RNA was precipitated by adding isopropanol (Duksan, Seoul, Republic of Korea) for 10 min at room temperature. The RNA was pelleted via centrifugation at 12,000× *g* for 10 min at 4 °C and washed with 75% cold ethanol (Sigma-Aldrich, St. Louis, MO, USA). The RNA pellet was dried for 10 min at room temperature and dissolved in diethyl-pyrocarbonate-treated water (DEPC water; Biosesang, Seongnam, Republic of Korea). The RNA concentration and purity were quantified using a NanoDrop spectrophotometer (Thermo Fisher Scientific, Rockford, IL, USA).

For cDNA synthesis, 1 µg of extracted RNA was mixed with Oligo DT primers (TAKARA, Tokyo, Japan) and dNTPs (TAKARA, Tokyo, Japan) in RNase-Free distilled water (TAKARA, Tokyo, Japan) and boiled at 65 °C for 5 min. The mixture was mixed with reverse transcriptase (TAKARA, Tokyo, Japan), RNase inhibitor was added (TAKARA, Tokyo, Japan), and the mixture was boiled at 42 °C for 45 min and then 95 °C for 5 min in a thermal cycler (Bio-Rad Hercules, CA, USA).

#### 4.4.2. Protein Extraction

Cells (5 × 10^6^ cells per mL) were lysed in a radioimmunoprecipitation assay buffer supplemented with protease and phosphatase inhibitors (EzRIPA buffer kit; ATTO Corporation, Tokyo, Japan). Skin tissues were homogenized using a bead homogenizer (Allsheng Instrument, Hangzhou, China) at 6.0 m/s for 5 cycles (which ran for 40 s and were interrupted for 45 s) in EzRIPA buffer. Then, the cells or skins were incubated on ice for 10 min to facilitate cell lysis. After sonication, the samples were centrifuged at 14,000× *g* for 15 min at 4 °C; then, the supernatants were collected and the protein concentration was quantified using a bicinchoninic acid assay kit (Thermo Fisher Scientific, Rockford, IL, USA) according to the manufacturer’s instructions.

#### 4.4.3. Paraffin-Embedded Skin Blocks

Skin tissues were fixed in 4% paraformaldehyde (Sigma-Aldrich, St. Louis, MO, USA) for 48 h at 4 °C. The fixed tissues were dehydrated in increasing concentrations of ethanol, cleared in xylene (Duksan, Seoul, Republic of Korea), and embedded in paraffin using a tissue processor (Leica, Wetzlar, Germany). The paraffin-embedded tissue blocks were sectioned into 7 μm slices using a microtome (Thermo Fisher Scientific, Rockford, IL, USA). The sections were mounted on coated microscope slides (Muto pure chemical Co., Ltd., Tokyo, Japan) and baked at 60 °C for 24 h to enhance tissue adhesion.

### 4.5. Quantitative Real-Time Polymerase Chain Reaction (qRT-PCR)

qRT-PCR was performed using 5 µL of ROX plus SYBR green premix (TAKARA), 0.8 µL of each reverse and forward primer (Table S1), 1.7 µL of DW, and 2.5 µL of cDNA template in a 10 µL total volume. Amplification and melting curve analyses were conducted using a real-time PCR instrument (Thermo Fisher Scientific, Rockford, IL, USA). The qRT-PCR amplification was performed with an initial denaturation step of 95 °C for 10 min, followed by 40 cycles of 95 °C for 15 s, 60 °C for 1 min, and 95 °C for 15 s. After the amplification, the melting analysis was performed at between 60 °C and 95 °C at a rate of increase of 0.075 °C/s. The gene expression level was analyzed using the comparative CT method (ΔΔCT). The mRNA level was normalized to *Actb* and expressed relative to the level in the first bar of each graph.

### 4.6. Western Blot

For the Western blot, 50 µg of proteins per sample was diluted in loading buffer (LDS buffer; Thermo Fisher Scientific, Rockford, IL, USA) and sample-reducing agent (Thermo Fisher Scientific, Rockford, IL, USA) and then denaturized at 70 °C for 10 min, and, afterwards, it was kept on ice for 10 min. To confirm the protein expression of HSP90, HIF-1α, or PI3K, 8% sodium dodecyl sulfate (SDS)-polyacrylamide gel was used for electrophoresis. For the analysis of VEGF, AKT, pAKT, ERK1/2, pERK1/2, or β-actin, 10% SDS-polyacrylamide gel was used. Additionally, VEGFR2 expression was analyzed with 3–8% Tris-Acetate gel (Invitrogen, Rockford, IL, USA). The denatured protein was electrophoresed using MOPS buffer (Invitrogen, Rockford, IL, USA) for 8% or 10% SDS-polyacrylamide or Tris-Acetate SDS running buffer (Invitrogen, Rockford, IL, USA) for 3–8% Tris-Acetate gel (Invitrogen, Rockford, IL, USA) at 200 V and then electro-transferred (Semi-dry transfer system; ATTO Corporation, Tokyo, Japan) to the polyvinylidene fluoride (PVDF) membranes (Merck Millipore, Burlington, MA, USA) with 1 A for 10 min. The membranes were blocked for 1 h with 5% skim milk (LPS solution, Daejeon, Republic of Korea) in tris-buffered saline with 0.1% Tween 20 (TTBS; LPS solution, Daejeon, Republic of Korea) at room temperature. After being washed with TTBS, the primary antibody was diluted with TTBS in the appropriate proportions (Table S2) and the membranes were incubated overnight with primary antibody at 4 °C. Then, the membranes were washed with TTBS and incubated with horseradish peroxidase (HRP)-conjugated secondary antibody (Vector Laboratories, Burlingame, CA, USA) for 2 h at room temperature (Table S2). After incubation, the membranes were washed with TTBS and protein bands were detected using the ChemiDoc Imaging Systems (Bio-Rad, Hercules, CA, USA) after a 3 min reaction using enhanced chemiluminescence solution (Cytiva^TM^, Marlborough, MA, USA). All protein bands were quantified using ImageJ software (National Institutes of Health, NIH, Maryland, MD, USA). The expression of β-actin was used as the endogenous control and expressed as the fold change relative to the first bar in each graph.

### 4.7. Angiogenesis Assay

#### 4.7.1. Wound Migration Assay

ECs were seeded at a density of 5 × 10^3^ cells per well in a 12-well plate (LPS solution, Daejeon, Republic of Korea) and allowed to attach overnight. Senescent ECs were prepared as described above (see Section 4.2.3). Then, the cells were scratched in an area at the center of each well with a sterile 200 μL pipette tip (Axygen, Somerville, MA, USA), generating a “wound” of uniform width. Then, scratched cells were treated with CM. Cell migration was measured 48 h after wounding using Axio Observer apparatus (Zeiss, Jena, Germany). Images were captured at 4× magnification using a digital camera (Olympus, Tokyo, Japan). The distance between the edges of the wound was measured using Image J software (NIH, Maryland, MD, USA) [92].

#### 4.7.2. Tube Formation Assay

Matrigel (BD Bioscience, Franklin Lakes, NJ, USA) was thawed overnight at 4 °C and kept on ice until use. Matrigel was added to a 12-well plate (LPS solution, Daejeon, Republic of Korea) and allowed to polymerize for 30 min at 37 °C. ECs were seeded onto the Matrigel-coated wells at a density of 5 × 10^3^ cells per well, and senescence was induced using H_2_O_2_ as previously described (see Section 4.2.3). After treatment with CM for 48 h, tube formation was observed with Axio Observer apparatus (Zeiss, Jena, Germany), and images were captured using a digital camera (Olympus, Tokyo, Japan). The number of tube-like structures was quantified using Image J software (NIH, Maryland, MD, USA) [92,93].

#### 4.7.3. Proliferation Assay

ECs were seeded in 96-well plates (LPS solution, Daejeon, Republic of Korea) at a density of 1 × 10^3^ cells per well and allowed to adhere for 24 h. The cell proliferation of the ECs in response to the PDLLA, PN, or CaHA treatment was confirmed using a cell counting kit (Transgen Biotech Co., Ltd., Beijing, China) once the in vitro model was manufactured, as mentioned in Section 4.2.3. Cell counting kit solution was mixed with serum-free medium (1:9, *v*/*v*), the mixture was added to each well, and the cells were incubated for 4 h at 37 °C. The optical density was measured at 450 nm using a microplate reader (Multiskan SkyHigh Photometer; Thermo Fisher Scientific, Rockford, IL, USA).

### 4.8. Enzyme-Linked Immunosorbent Assay (ELISA)

Flat-bottomed 96-well plates (LPS solution, Daejeon, Republic of Korea) were coated with 0.6% sodium bicarbonate (Sigma-Aldrich, St. Louis, MO, USA) and 0.3% sodium carbonate (Sigma-Aldrich, St. Louis, MO, USA) in DW and incubated overnight at 4 °C. After being washed with PBS containing 0.1% Tween 20 (TPBS), the plates were blocked with 5% skim milk (LPS solution, Daejeon, Republic of Korea) in PBS for 4 h at room temperature. The wells were washed with TPBS, 80 μg protein samples were added to each well, and the plates were incubated overnight at 4 °C with shaking. The plates were then washed and the appropriate detection antibody was added (Table S2) and incubated at 4 °C overnight with shaking. After being rinsed with TPBS, HRP-conjugated antibody solution was added (Table S2) and the plates were incubated at room temperature for 2 h. After being washed, the HRP substrate solution (3,3′,5,5′-tetramethylbenzidine; Sigma-Aldrich, St. Louis, MO, USA) was added and the plates were incubated for 15 min at room temperature in the dark to allow for color development. The reaction was stopped with an equal volume of stop solution (2 M H_2_SO_4_; Sigma-Aldrich, St. Louis, MO, USA) and absorbance at 450 nm was measured using a microplate reader (Multiskan SkyHigh Photometer; Thermo Fisher Scientific, Rockford, IL, USA).

### 4.9. Histological Analysis

#### 4.9.1. Masson’s Trichrome Staining

Skin tissue sections were deparaffinized and rehydrated via sequential incubation in a series of xylene (Duksan, Seoul, Republic of Korea) and 70–100% gradient alcohols (Duksan, Seoul, Republic of Korea). The sections were then stained using a Masson’s trichrome staining kit (Scytek Laboratories, West Logan, UT, USA) according to the manufacturer’s instructions to identify collagen fibers. Briefly, the sections were incubated in Bouin solution at 60 °C for 1 h and washed with DW. The sections were then stained in a working weight solution of iron hematoxylin for 5 min, followed by a Biebrich scarlet acid fuchsin solution for 5 min. After being rinsed with DW, the sections were incubated with a phosphomolybdic-phosphotungstic acid solution for 12 min and, then, they were stained with aniline blue solution for 3 min at room temperature. The sections were then dehydrated in a 100–70% down-gradient series of alcohols, cleared in xylene (Duksan, Seoul, Republic of Korea), and mounted with coverslips using DPX mount solution (Sigma-Aldrich, St. Louis, MO, USA). The Masson’s trichrome-stained sections were examined under an optical microscope (Olympus, Tokyo, Japan) equipped with a slide scanner (Motic, Beijing, China). Collagen fibers were stained blue, while the cytoplasm and nuclei were stained red and black, respectively. The collagen fiber density was measured using the Image J software (NIH, Maryland, MD, USA) to determine the collagen portion of the whole tissue and its relative proportion to the young mouse tissue.

#### 4.9.2. Herovici’s Staining

Deparaffinized and rehydrated tissue sections were stained using a Herovici’s stain kit (Scytek Laboratories) according to the manufacturer’s instructions. This staining protocol differentiates mature collagen from newly synthesized collagen. Briefly, the sections were incubated with Weigert’s iron hematoxylin to stain the nuclei for 8 min at room temperature. After being washed with tap water and DW, the sections were stained with Herovici solution for 2 min at room temperature and dehydrated with 70–100% gradient alcohols. The sections were then cleared in xylene (Duksan, Seoul, Republic of Korea) and mounted using coverslips and DPX mounting medium (Sigma-Aldrich, St. Louis, MO, USA). The slides were examined under a microscope (Olympus, Tokyo, Japan) and images were captured using a slide scanner (Motic, Beijing, China). Mature collagen was stained red and newly synthesized collagen was stained blue [70,71]. The density of mature collagen (red–yellow area) or newly synthesized collagen (blue area) was measured using Image J software (NIH, Maryland, MD, USA).

### 4.10. Measurement of Dermal Thickness

To measure the dermal thickness, the skin tissues were stained with hematoxylin and eosin. Briefly, the sections were deparaffinized in xylene and rehydrated through a series of graded alcohols (70–100%). The sections were then stained with hematoxylin solution (KPNT, Cheongju, Republic of Korea) for 1 min and washed in running tap water 3 min. The sections were then incubated with ammonia water (KPNT, Cheongju, Republic of Korea) for 10 s, immersed in eosin solution (KPNT, Cheongju, Republic of Korea) for 1 min, and washed with running water, dehydrated through graded alcohols (100–70%), cleared in xylene, and mounted with a coverslip in mounting medium (DPX solution; Sigma-Aldirch, St. Louis, MO, USA). The stained sections were imaged using a slide scanner (Motic, Beijing, China). Dermal thickness was measured using ImageJ software (NIH, Maryland, MD, USA). Briefly, the thickness of the dermis was measured at five randomly selected locations per section, and five sections per sample were analyzed. The data obtained were expressed as the mean dermal thickness per sample.

### 4.11. Statistical Analysis

Statistical analysis was performed using SPSS software (version 22; IBM Corporation, Armonk, NY, USA). Data are presented as mean ± standard deviation. Statistical significance was analyzed using a Kruskal–Wallis test and a post hoc Mann–Whitney U test. *p* values less than 0.05 were considered statistically significant. Statistical significance was determined using the following pairwise comparisons:

*: first bar vs. second bar;
$: second bar vs. third, fourth, or fifth bar;
#: third bar vs. fourth or fifth bar.

## 5. Conclusions

In conclusion, PDLLA, PN, and CaHA stimulate the expression of HSP90, HIF-1α, and VEGF, which, in turn, upregulate PI3K, pAKT/AKT, and pERK1/2/ERK1/2, leading to increased angiogenesis and decreased oxidative stress in aged skin. When PDLLA was injected into the skin of old mice, the expression levels of all three TGF-β isoforms increased and the density of collagen fibers increased. Even though angiogenesis could lead to skin rejuvenation by decreasing oxidative stress, research into the rejuvenation effect of dermal fillers has previously focused on collagen or elastin fiber synthesis. The present study suggested that PDLLA, PN, and CaHA could result in increased angiogenesis, accompanied by an increased abundance of collagen fibers. Moreover, the present study suggested that one possible pathway to increase angiogenesis is HSP90/HIF-1α/VEGF. Overall, PDLLA, PN, and CaHA fillers could be used for skin rejuvenation via increasing angiogenesis in aged skin.

## Data Availability

Not applicable.

## Supplementary Materials

The following supporting information can be downloaded at: https://www.mdpi.com/article/10.3390/ijms24097986/s1.
