# Supplementary material for: Poly-D,L-Lactic Acid Stimulates Angiogenesis and Collagen Synthesis in Aged Animal Skin
Source: Int J Mol Sci. 2023 Apr 28;24(9):7986. doi: 10.3390/ijms24097986 (PMC10178436; doi:10.3390/ijms24097986)
Supplement: Supplementary file 1 [file ijms-24-07986-s001.zip › ijms-2361888-supplementary.pdf]

International Journal of
Molecular Sciences

MDPI

Article

Poly-D, L-lactic acid stimulates angiogenesis and collagen synthesis in aged animal skin

Seyeon Oh [1,†], Suk Bae Seo [2,†], Gunpoong Kim [3], Sosorburam Batsukh [1,4], Kuk Hui Son [5,*] and Kyunghee Byun [1,4,*]

[1] Functional Cellular Networks Laboratory, Graduate School and Lee Gil Ya Cancer and Diabetes Institute, College of Medicine, Gachon University, Incheon 21999, Korea
[2] SeoAh Song Dermatologic Clinic, Seoul 05557, Korea
[3] VAIM Co., LTD, Okcheon, 29055, Korea
[4] Department of Anatomy & Cell Biology, College of Medicine, Gachon University, Incheon 21936, Korea
[5] Department of Thoracic and Cardiovascular Surgery, Gachon University Gil Medical Center, Gachon University, Incheon 21565, Korea
* Correspondence: dr632@gilhospital.com (K.H.S.); Tel.: +82-32-460-3666 (K.H.S.); khbyun1@gachon.ac.kr (K.B.); Tel.: +82-32-899-6511 (K.B.)

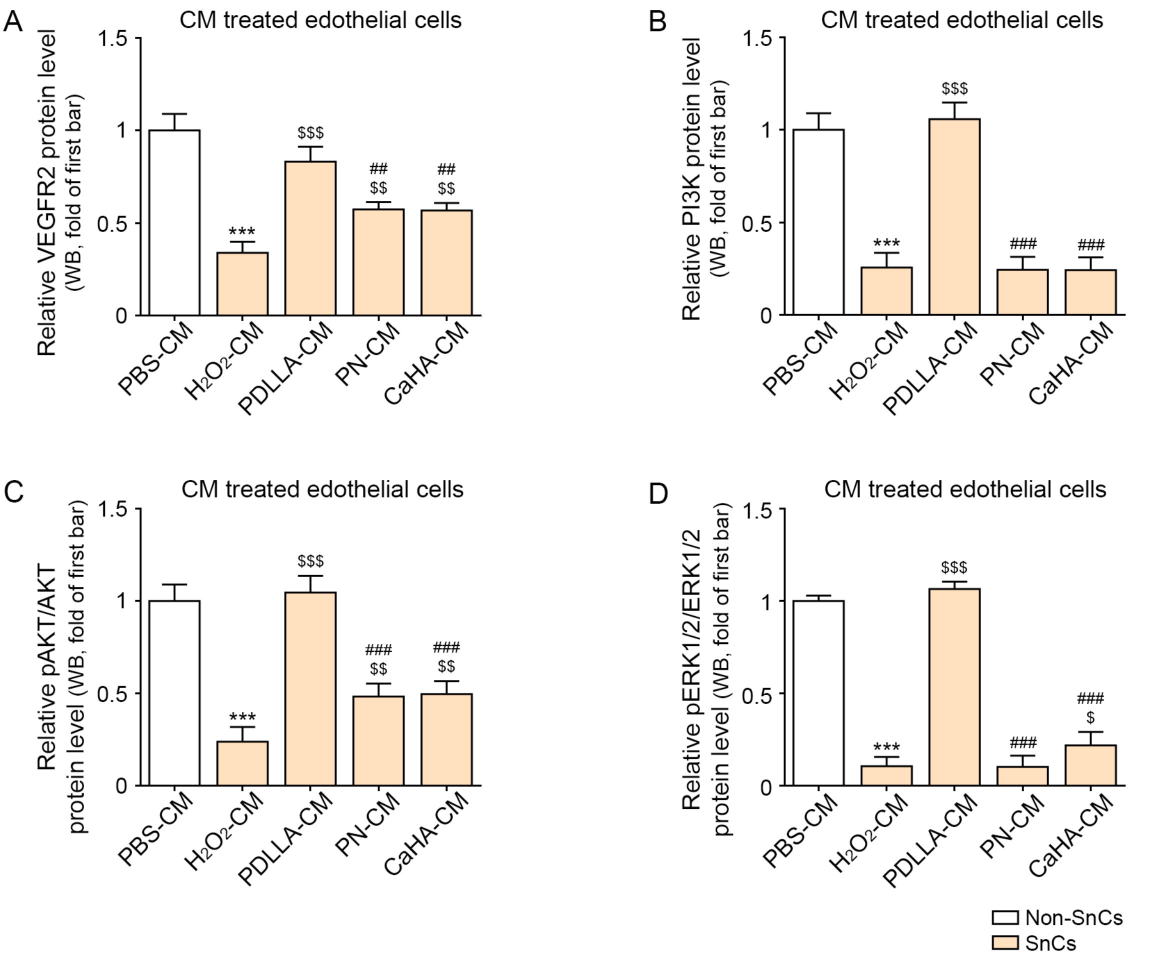

Figure S1. Upregulation of VEGFR2, PI3K, pAKT, and pERK1/2 in senescent endothelial cells exposed to CM from PDLLA-treated senescent macrophages. (**A–D**) Quantitation of results shown in Figure 2B. Expression of VEGFR2, PI3K, pAKT/AKT, and pERK1/2/ERK1/2 was lower in senescent endothelial cells exposed to H_2O_2/PBS-CM and higher in endothelial cells exposed to H_2O_2/PDLLA-CM, PN-CM, or CaHA-CM. To correct for differences in protein loading, the quantification of the Western blot was normalized using β-actin as a loading control protein. For each blot, the values were expressed relative to the mean of the first group. Data are presented as mean ± SD (*n*=3/group). ***, $p < 0.001$, first bar vs. second bar; $, $$, and $$$, $p < 0.05$, $p < 0.01$, and $p < 0.001$, second bar vs. third, fourth and fifth bar; ## and ###, $p < 0.01$ and $p < 0.001$, third bar vs. fourth and fifth bar. AKT, protein kinase B; β-actin, beta-actin; CaHA, calcium hydroxyapatite; CM, conditioned medium; ERK1/2, extracellular signal-regulated kinase 1/2; kDa, kilodalton; MW, molecular weight; pAKT, phosphorylated AKT; PBS, phosphate-buffered saline; PDLLA, poly-D, L-lactic acid; pERK1/2, phosphorylated ERK1/2; PI3K, phosphoinositide 3-kinase; PN, polynucleotide; SD, standard deviation; SnCs, senescent cells; VEGFR2, vascular endothelial growth factor receptor 2; WB, Western blot.

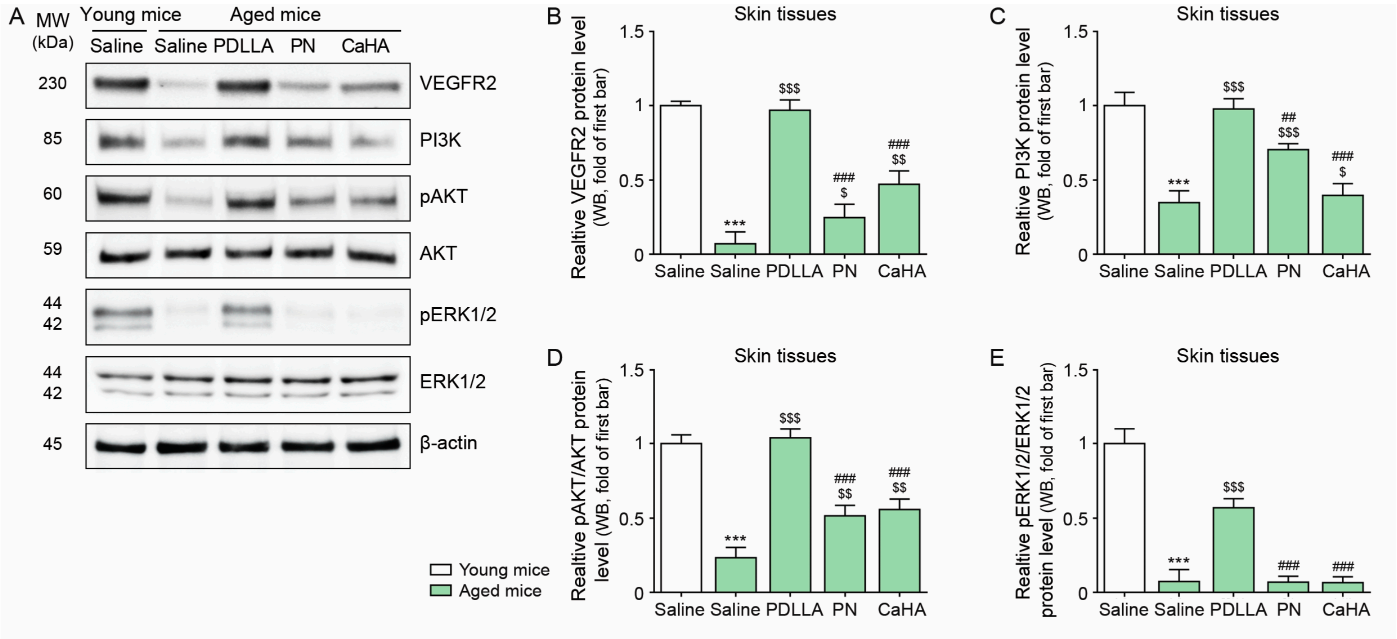

Figure S2. Upregulation of VEGFR2, PI3K, pAKT, pERK1/2 via PDLLA in aged skin. (**A**) Expression of VEGFR2, PI3K, AKT, pAKT, ERK1/2, and pERK1/2 in aged skin was confirmed via Western blot. (**B–E**) Quantitation of results shown in (A). VEGFR2, PI3K, pAKT/AKT, and pERK1/2/ERK1/2 levels were lower in old mice injected with saline and higher in old mice injected with PDLLA, PN, or CaHA. To correct for differences in protein loading, the quantification of the Western blot was normalized using beta-actin as a loading control protein. For each blot, the values were expressed relative to the mean of the first group. Data are presented as mean ± SD (n=3/group). **, $p < 0.001$, first bar vs. second bar; \$, \$\$, and \$\$\$, $p < 0.05$, $p < 0.01$, and $p < 0.001$, second bar vs. third, fourth and fifth bar; ## and ###, $p < 0.01$ and $p < 0.001$, third bar vs. fourth and fifth bar. AKT, protein kinase B; β-actin, beta-actin; CaHA, calcium hydroxyapatite; ERK1/2, extracellular signal-regulated kinase 1/2; kDa, kilodalton; MW, molecular weight; pAKT, phosphorylated AKT; PDLLA, poly-D, L-lactic acid; pERK1/2, phosphorylated ERK1/2; PI3K, phosphoinositide 3-kinase; PN, polynucleotide; SD, standard deviation; VEGFR2, vascular endothelial growth factor receptor 2; WB, Western blot.

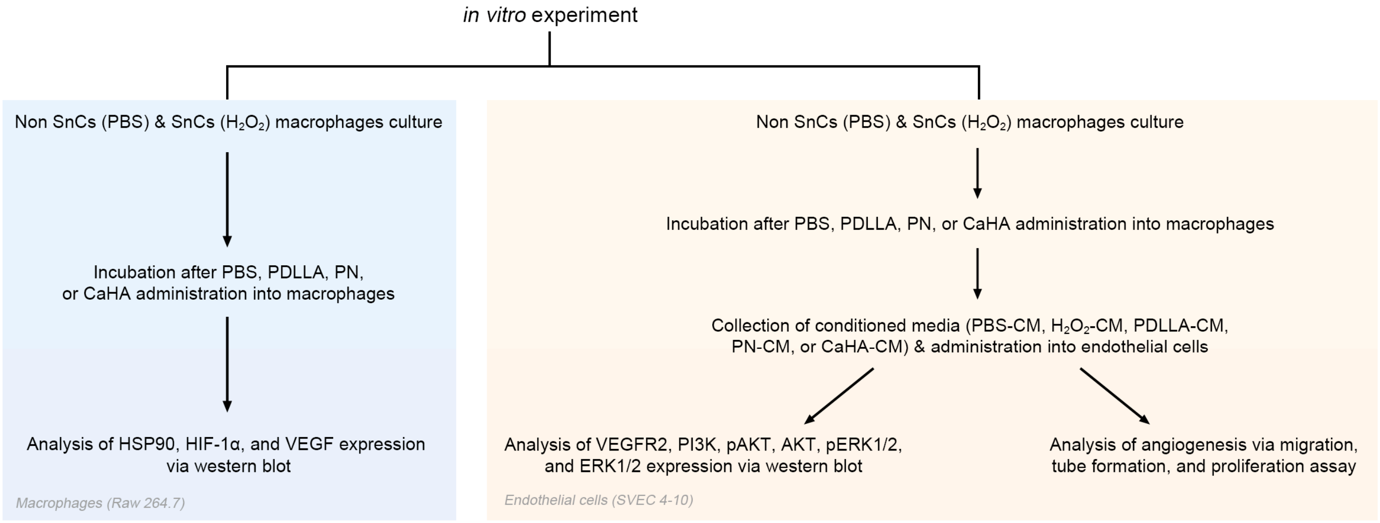

Figure S3. The schematic diagram of the *in vitro* experiment process. AKT, protein kinase B; CaHA, calcium hydroxyapatite; CM, conditioned medium; ERK1/2, extracellular signal-regulated kinase 1/2; HIF-1α, hypoxia-inducible factor-1 alpha; HSP90, heat shock protein 90; PDLLA, poly-D, L-lactic acid; PN, polynucleotide; phosphorylated AKT; pERK1/2, phosphorylated ERK1/2; PBS, phosphate-buffered saline; PI3K, phosphoinositide 3-kinase; ROS, reactive oxidative species; TGF-β, tumor growth factor-beta; VEGF, vascular endothelial growth factor; VEGFR2, vascular endothelial growth factor receptor 2.

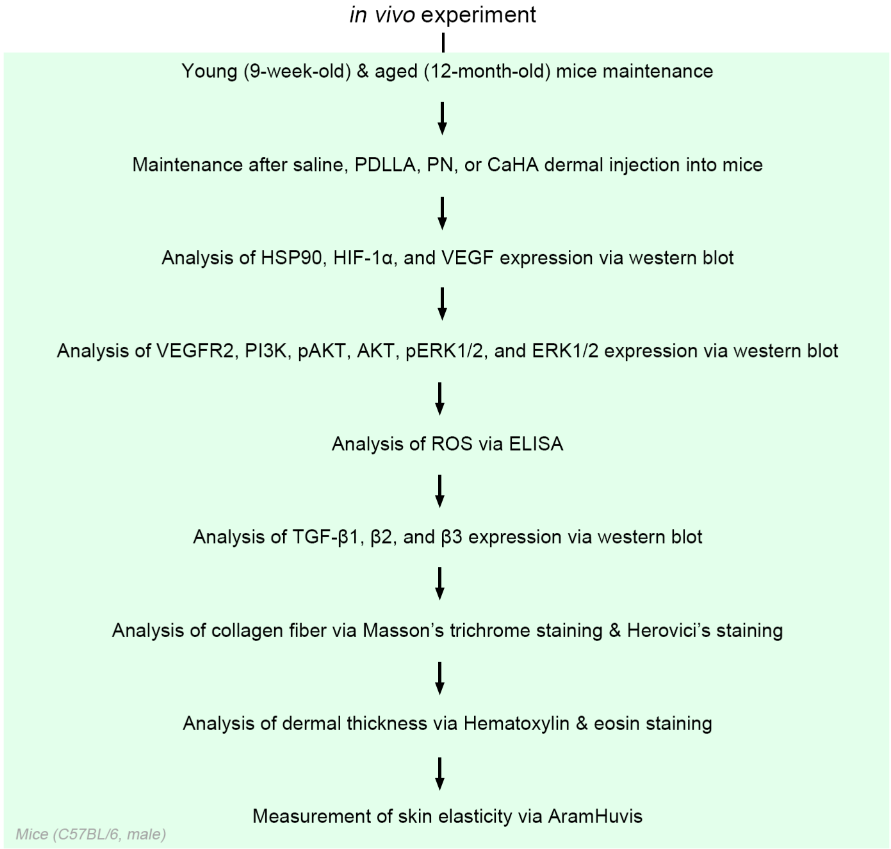

Figure S4. The schematic diagram of the *in vivo* experiment process. AKT, protein kinase B; CaHA, calcium hydroxyapatite; ERK1/2, extracellular signal-regulated kinase 1/2; HIF-1α, hypoxia-inducible factor-1 alpha; HSP90, heat shock protein 90; PDLLA, poly-D, L-lactic acid; PN, polynucleotide; phosphorylated AKT; pERK1/2, phosphorylated ERK1/2; PI3K, phosphoinositide 3-kinase; ROS, reactive oxidative species; TGF-β, tumor growth factor-beta; VEGF, vascular endothelial growth factor; VEGFR2, vascular endothelial growth factor receptor 2.

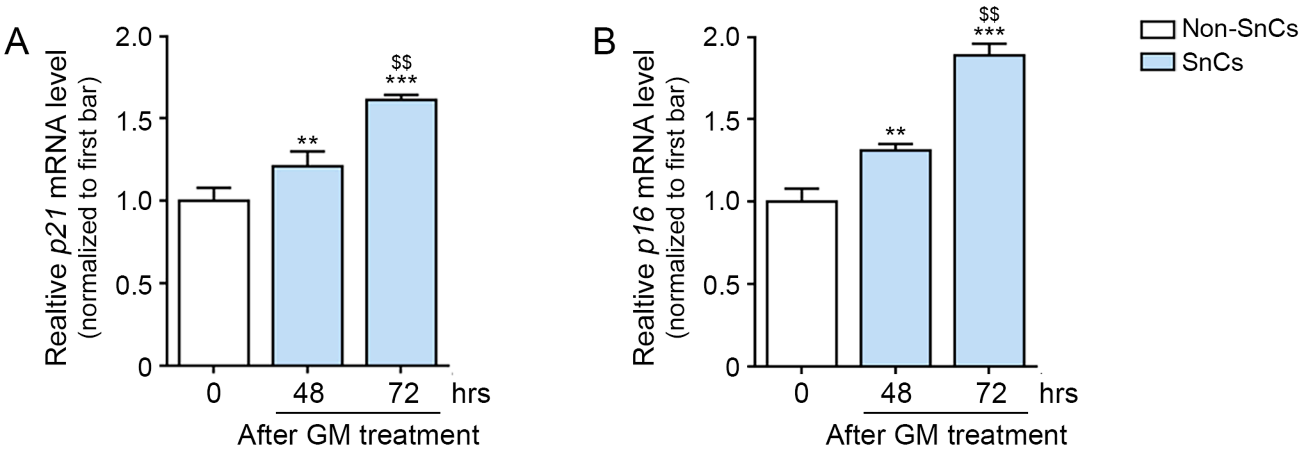

Figure S5. The optimal time to induce senescence in macrophages was determined. (**A and B**) To obtain the optimal time to induce senescence in macrophages, the mRNA expression of p21 and p16 (senescence markers) was measured via qRT-PCR. The mRNA expression of p21 and p16 was increased after treatment of macrophages with 100 μM of H_2O_2 for 2 hours and GM for 48 or 78 hours. The results showed more senescence at 78 hours than at 48 hours. Data were normalized to Actb, and expression levels were reported according to the comparative CT method (ΔΔCT) relative to the first bar in the graph. Data are presented as the mean ± SD (*n*=3/group). **, *p* < 0.01, first bar vs. second or third bar; $$, *p* < 0.01, second bar vs. third bar.

Table S1. List of primers used in this study.

Gene	Primers	
Actb	Forward	5'-AGAAGGACTCCTATGTGGGTGA-3′
	Reverse	5'-GGCATAGAGGTCTTTACGGATG-3′
p21	Forward	5′-GAGAACGGTGGAACTTTGACTT-3′
	Reverse	5′-CTCAGACACCAGAGTGCAAGAC-3′
p16	Forward	5′-CAACGCACCGAATAGTTACG-3′
	Reverse	5′-ATCTATGCGGGCATGGTTACT-3′

Table S2. List of antibodies used in this study.

Antibody	Company	Catalog no.	Dilution rate	
			WB	ELISA
HSP90	GeneTex	GTX109753	1:5,000	
HIF-1α	Santa Cruz Biotechnology	sc-53546	1:200	
VEGF	FineTest	FNab09391	1:1,000	
VEGFR2	Cell Signaling Technology	2479	1:1,000	
PI3K	Santa Cruz Biotechnology	sc-376112	1:200	
pAKT	Cell Signaling Technology	4060	1:1,000	
AKT	BD Biosciences	610860	1:1,000	
pERK1/2	Cell Signaling Technology	9101	1:1,000	
ERK1/2	Cell Signaling Technology	9102	1:1,000	
4HNE	Bioss Antibodies	bs-6313R		1:500
TGF-β1	Abcam	ab64715	1:4,000	
TGF-β2	FineTest	FNab08639	1:500	
TGF-β3	FineTest	FNab08640	1:500	
COL1A1	Santa Cruz Biotechnology	sc-293182		1:500
COL3A1	Bioss Antibodies	bs-0549R		1:500
β-actin	Cell Signaling Technology	4967	1:1,000	

4HNE, 4-hydroxy-2-nonenal; AKT, protein kinase B; COL1A1, collagen type 1a1; COL3A1, collagen type 3a1; ERK1/2, extracellular signal-regulated kinase 1/2; β-actin, beta-actin; HIF-1α, hypoxia-inducible factor-1 alpha; HSP90, heat shock protein 90; pAKT, phosphorylated AKT; pERK1/2, phosphorylated ERK1/2; PI3K, phosphoinositide 3-kinase; TGF-β, tumor growth factor-beta; VEGF, vascular endothelial growth factor; VEGFR2, VEGF receptor 2.

Disclaimer/Publisher's Note: The statements, opinions and data contained in all publications are solely those of the individual author(s) and contributor(s) and not of MDPI and/or the editor(s). MDPI and/or the editor(s) disclaim responsibility for any injury to people or property resulting from any ideas, methods, instructions or products referred to in the content.
